# Supplementary material for: Psoas Muscle Index as an Independent Predictor of Survival in Patients with Hepatocellular Carcinoma Receiving Systemic Targeted Therapy
Source: Cancers (Basel). 2025 Jan 10;17(2):209. doi: 10.3390/cancers17020209 (PMC11763421; doi:10.3390/cancers17020209)

Figure S2. Kaplan–Meier curves for cumulative survival in patients treated with sorafenib or lenvatinib divided by the optimal PMI cut-off value for (a) women (>2.86 and ≤2.86 cm²/m²) and (b) men (>3.55 and ≤3.55 cm²/m²).

(a) Female

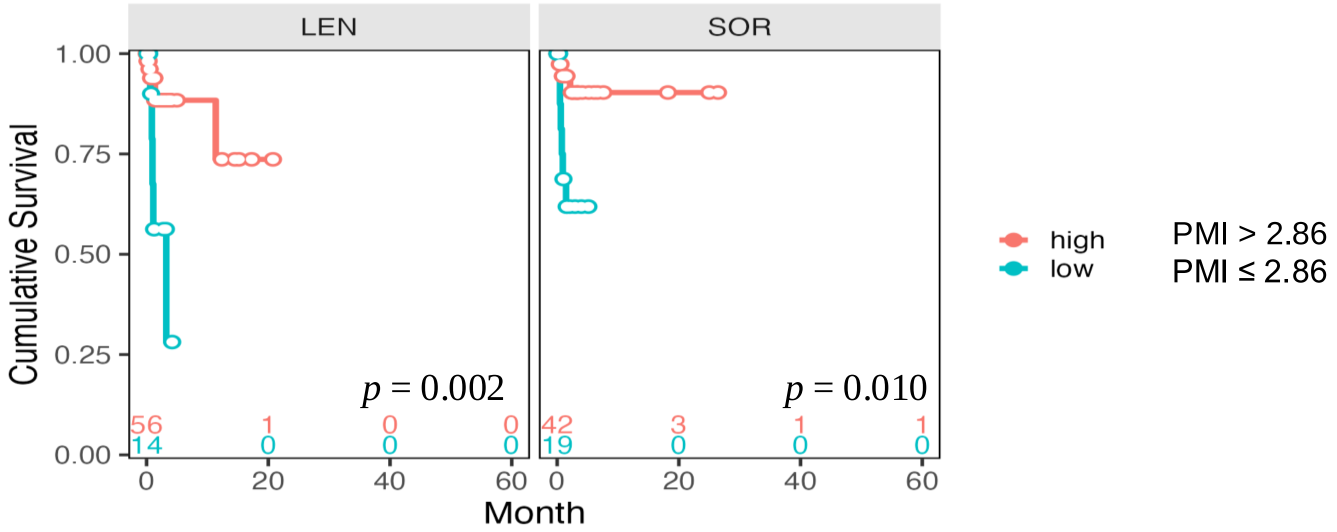

(a) Male

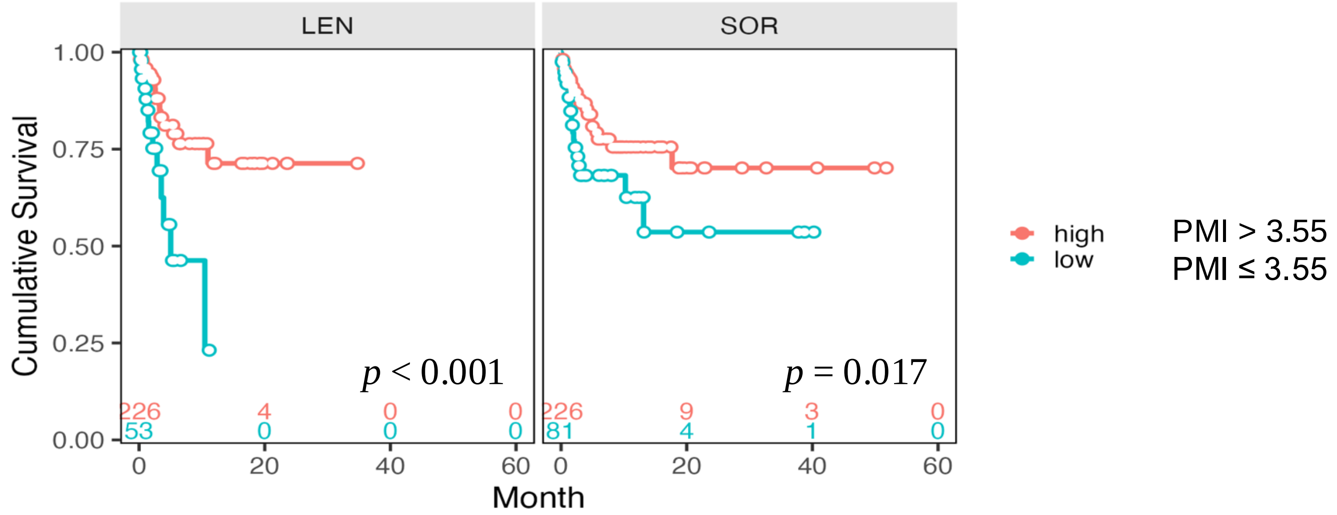

Supplement: Supplementary file 1 [file cancers-17-00209-s001.zip › FigureS2.pdf]
