# Supplementary material for: Psoas Muscle Index as an Independent Predictor of Survival in Patients with Hepatocellular Carcinoma Receiving Systemic Targeted Therapy
Source: Cancers (Basel). 2025 Jan 10;17(2):209. doi: 10.3390/cancers17020209 (PMC11763421; doi:10.3390/cancers17020209)
Supplement: Supplementary file 1 [file cancers-17-00209-s001.zip › TableS1.pdf]

Table S1. Univariate and multivariate analyses of predictors for survival in patients treated with sorafenib or lenvatinib using the time-dependent Cox proportional hazards model

	HR (95%CI)	*p* value
Univariate analysis		
PMI (cm^2/m^2)	0.773 (0.673–0.889)	< 0.001
SMI (cm^2/m^2)	0.922 (0.899–0.946)	<0.001
AFP ($\times 10^3$ ng/mL)	1.001 (1.001–1.002)	< 0.001
ALBI score	3.635 (2.775–4.761)	< 0.001
Multivariate analysis (adjusted for AFP and ALBI score)		
PMI (cm^2/m^2)	0.812 (0.707–0.932)	0.003
SMI (cm^2/m^2)	0.946 (0.924–0.968)	< 0.001

All the variables were dealt with as time-varying covariates. HR, hazard ratio; CI, confidence interval; PMI, psoas muscle index; SMI, skeletal muscle index; AFP, alpha-fetoprotein; ALBI score, albumin-bilirubin score;
