# Supplementary material for: Psoas Muscle Index as an Independent Predictor of Survival in Patients with Hepatocellular Carcinoma Receiving Systemic Targeted Therapy
Source: Cancers (Basel). 2025 Jan 10;17(2):209. doi: 10.3390/cancers17020209 (PMC11763421; doi:10.3390/cancers17020209)
Supplement: Supplementary file 1 [file cancers-17-00209-s001.zip › TableS2.pdf]

Table S2. Comparison of clinical characteristics and adverse events between lower and higher PMI groups

	Lower PMI group (n = 32)	Higher PMI group (n = 182)	*p* value
Age (years)	77 [70.25, 80]	73 [66.25, 78]	0.100
Sex (male/female)	25/7	146/36	0.812
Etiology (HBV/HCV/others)	9/9/14	35/54/93	0.510
Drug(AB/DT/LEN/SOR/CAB/RAM/REG)	38/5/66/63/5/3/1	5/0/10/16/1/0/0	0.744
BCLC stage (A/B/C)	1/12/19	12/73/97	0.780
ALBI score	-2.220 [-2.675, -1.910]	-2.420 [-2.780, -2.115]	0.134
ECOG PS (0/1/2/3)	24/6/2/0	138/33/10/1	0.950
AFP ($\times 10^3$ ng/mL)	0.167 [0.012, 1.176]	0.061 [0.006, 1.521]	0.385
General fatigue (0/1/2/≥3)	19/5/7/1	99/32/43/8	0.973
Appetite loss (0/1/2/≥3)	15/5/10/2	92/28/53/9	0.955
Diarrhea (0/1/2/≥3)	27/3/2/0	139/29/11/3	0.796
Hypothyroidism (0/1/2/≥3)	29/0/3/0	143/15/24/0	0.218
Hand-foot syndrome (0/1/2/≥3)	28/1/1/2	135/21/25/1	0.016
Proteinuria (0/1/2/≥3)	28/1/2/1	130/14/19/19	0.382
Hypertension (0/1/2/≥3)	23/2/6/1	119/9/34/20	0.623

PMI, psoas muscle index; HCV, hepatitis C virus; AB, atezolizumab/bevacizumab; DT, tremelimumab/durvalumab; LEN, lenvatinib; SOR, sorafenib; CAB, cabozantinib; RAM, ramcirumab; REG, regorafenib; BCLC stage, Barcelona Clinic Liver Cancer stage; ECOG, Eastern cooperative oncology group; PS, performance status; AFP, alpha-fetoprotein;
