# Supplementary figures and images for: Psoas Muscle Index as an Independent Predictor of Survival in Patients with Hepatocellular Carcinoma Receiving Systemic Targeted Therapy
Source: Cancers (Basel). 2025 Jan 10;17(2):209. doi: 10.3390/cancers17020209 (PMC11763421; doi:10.3390/cancers17020209)

Figure S1. Patient flow in this study

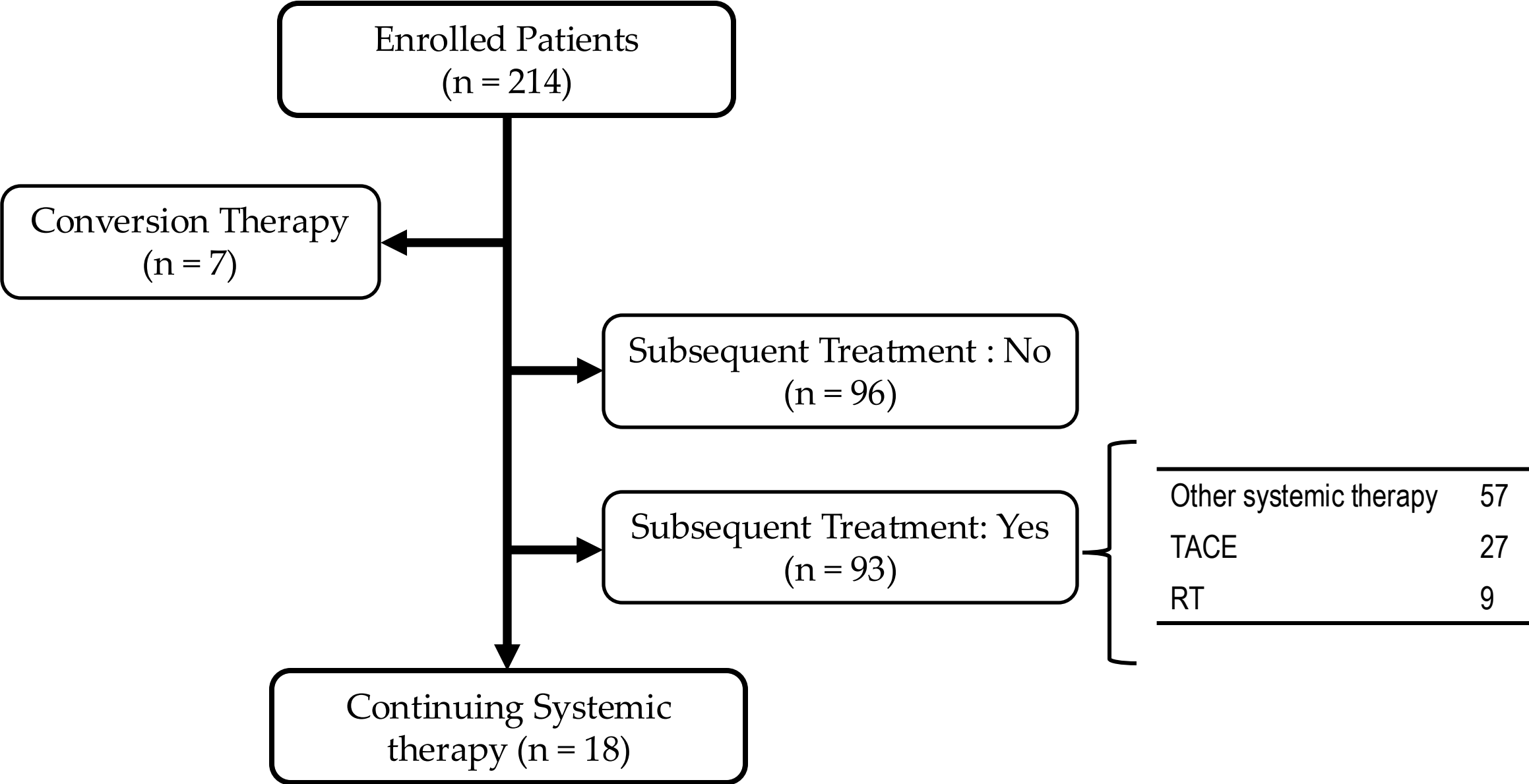

Supplement: Supplementary file 1 [file cancers-17-00209-s001.zip › FigureS1.pdf]
